# Proctitis and Other Gastrointestinal Manifestations in Mpox Disease: A Systematic Review and Meta‐Analysis

**DOI:** 10.1002/jgh3.70190

**Published:** 2025-05-28

**Authors:** Prakasini Satapathy, Abhay M. Gaidhane, Nasir Vadia, Soumya V. Menon, Kattela Chennakesavulu, Rajashree Panigrahi, Sanjit Sah, Suraj Tiwari, S. Govinda Rao, Khang Wen Goh, Rachana Mehta, Muhammed Shabil, Mahendra Singh, Ganesh Bushi

**Affiliations:** ^1^ Center for Global Health Research, Saveetha Medical College and Hospital, Saveetha Institute of Medical and Technical Sciences Saveetha University Chennai India; ^2^ Faculty of Data Science and Information Technology INTI International University Nilai Malaysia; ^3^ Jawaharlal Nehru Medical College, and Global Health Academy, School of Epidemiology and Public Health Datta Meghe Institute of Higher Education Wardha India; ^4^ Department of Pharmaceutical Sciences, Marwadi University Research Center, Faculty of Health Sciences Marwadi University Rajkot Gujarat India; ^5^ Department of Chemistry and Biochemistry, School of Sciences JAIN (Deemed to Be University) Bangalore Karnataka India; ^6^ Department of Chemistry Sathyabama Institute of Science and Technology Chennai Tamil Nadu India; ^7^ Department of Microbiology, IMS and SUM Hospital Siksha ‘O’ Anusandhan (Deemed to Be University) Bhubaneswar Odisha India; ^8^ Department of Paediatrics, Dr. D. Y. Patil Medical College Hospital and Research Centre Dr. D. Y. Patil Vidyapeeth (Deemed‐to‐Be‐University) Pune Maharashtra India; ^9^ Department of Public Health Dentistry, Dr. D.Y. Patil Dental College and Hospital Dr. D.Y. Patil Vidyapeeth (Deemed‐to‐Be‐University) Pune Maharashtra India; ^10^ SR Sanjeevani Hospital Siraha Nepal; ^11^ Centre for Research Impact and Outcome, Chitkara University Institute of Engineering and Technology Chitkara University Rajpura Punjab India; ^12^ Division of Research and Innovation Uttaranchal University Dehradun India; ^13^ Department of Data Science Gokaraju Rangaraju Institute of Engineering and Technology Hyderabad Telangana India; ^14^ Faculty of Mathematics and Natural Sciences Universitas Negeri Padang Padang Indonesia; ^15^ Clinical Microbiology, RDC Manav Rachna International Institute of Research and Studies Faridabad Haryana India; ^16^ University Center for Research and Development Chandigarh University Mohali Punjab India; ^17^ Medical Laboratories Techniques Department AL‐Mustaqbal University Hillah Babil Iraq; ^18^ Department of Biotechnology Graphic Era (Deemed to Be University) Dehradun India; ^19^ Graphic Era Hill University Dehradun India; ^20^ School of Pharmaceutical Sciences Lovely Professional University Phagwara India

**Keywords:** gastrointestinal manifestations, meta‐analysis, monkeypox virus, Mpox, proctitis, systematic review

## Abstract

**Background:**

Mpox, caused by the monkeypox virus (MPXV), is primarily recognized for its dermatologic and systemic symptoms. However, emerging evidence suggests a significant prevalence of gastrointestinal (GI) manifestations, particularly proctitis, diarrhea, nausea, vomiting, and abdominal pain. Despite the growing clinical recognition of these symptoms, their epidemiology and impact remain poorly understood. This systematic review and meta‐analysis aim to quantify the prevalence of GI manifestations in Mpox patients and assess their clinical significance.

**Methods:**

A systematic review following PRISMA guidelines was conducted across PubMed, Embase, and Web of Science, including quantitative studies published up until October 2024 that reported GI manifestations in Mpox patients. Screening and data extraction were performed using Nested Knowledge software, and study quality was assessed using the Newcastle–Ottawa Scale. Meta‐analysis was conducted using R version 4.4, with heterogeneity evaluated via the *I* ^2^ statistic. Sensitivity analyses and publication bias were assessed using Doi plots and the Luis Furuya‐Kanamori (LFK) index.

**Results:**

Out of 1229 records, 33 studies met the eligibility criteria, yielding a pooled prevalence of proctitis in Mpox patients at 24.75% (95% CI: 18.93%–31.04%) across 5878 participants, with high heterogeneity (*I* ^2^ = 94.8%). The prediction interval for proctitis ranged from 1.46% to 61.76%. The pooled prevalence of other GI manifestations was 30.45% (95% CI: 18.27%–44.14%) across 2237 participants, with significant heterogeneity (*I* ^2^ = 95.2%) and a prediction interval ranging from 0.00% to 85.28%. Sensitivity analyses confirmed the stability of these estimates, while publication bias was indicated by LFK index values exceeding 2.77.

**Conclusions:**

This meta‐analysis highlights the substantial burden of GI manifestations in Mpox, particularly proctitis, with considerable variability across studies. The findings underscore the need for standardized diagnostic criteria and increased clinical recognition of GI symptoms in Mpox management. Further research into the underlying pathophysiology and integrating GI symptom assessment into Mpox surveillance and treatment strategies could enhance diagnostic accuracy and patient care outcomes.

## 1 Introduction

Mpox, caused by the monkeypox virus (MPXV), has emerged as a global public health concern following recent outbreaks beyond traditionally endemic regions [1]. While Mpox is primarily recognized for its characteristic skin lesions and systemic symptoms, increasing evidence suggests that gastrointestinal (GI) manifestations, particularly proctitis, are clinically significant [2]. Proctitis, characterized by inflammation of the rectal mucosa, is frequently reported among Mpox patients, especially in men who have sex with men (MSM) [3]. Other GI symptoms, such as diarrhea, rectal bleeding, nausea, vomiting, and abdominal pain, have also been observed. Despite these findings, the epidemiology and clinical significance of GI involvement in Mpox remain poorly understood. Given the potential morbidity associated with these symptoms, a comprehensive assessment of their prevalence and impact is critical for improving patient management and healthcare responses.

The mechanisms underlying Mpox‐related GI symptoms are not yet fully elucidated. Direct viral invasion of rectal mucosal tissues may trigger localized inflammation, leading to proctitis and related symptoms [4]. Systemic inflammatory responses, including cytokine‐mediated immune activation, could also contribute to GI dysfunction. Additionally, sexual transmission, particularly through receptive anal intercourse, has been proposed as a possible route for MPXV entry into rectal tissues, increasing the risk of proctitis [5, 6]. Furthermore, co‐infections with sexually transmitted infections (STIs) such as gonorrhea and chlamydia frequently accompany Mpox proctitis cases, complicating clinical presentation. These overlapping factors highlight the need for further investigation into the GI complications of Mpox to differentiate direct viral effects from co‐existing infections.

Although multiple studies have documented proctitis and other GI symptoms in Mpox patients, prevalence estimates vary widely. Some reports indicate that proctitis is among the most frequently observed symptoms, while others suggest a lower prevalence. This variability may stem from differences in study populations, diagnostic criteria, or regional differences in Mpox outbreaks. Unlike dermatologic and systemic manifestations, which are well documented, GI involvement remains underexplored, leading to uncertainty regarding its true burden and clinical implications. Additionally, differentiating Mpox‐associated proctitis from inflammatory bowel disease (IBD) or other viral infections presents diagnostic challenges. Given these uncertainties, a systematic review and meta‐analysis are needed to synthesize existing evidence, assess the burden of GI symptoms, and clarify their clinical significance.

This study aims to systematically review and analyze observational studies to determine the prevalence and clinical impact of proctitis and other GI manifestations in Mpox patients. By consolidating findings from diverse populations and study designs, this meta‐analysis will provide critical insights into the epidemiology of Mpox‐related GI symptoms. The results will refine clinical diagnostic criteria, guide patient management strategies, and inform public health policies. Given the global rise in Mpox cases and the emerging recognition of GI complications, this research is essential for optimizing healthcare responses and improving patient outcomes.

## 2 Methods

The systematic review adhered to the PRISMA guidelines, providing a clear and structured approach to assess and synthesize the available literature (Table S1) [7]. Furthermore, the study protocol was registered with PROSPERO under the ID CRD42024593152, helping to reduce the risk of redundant research and enhance the study's credibility by following predefined objectives and methodologies.

### 2.1 Eligibility

This systematic review includes studies that focus on patients diagnosed with Mpox and report GI manifestations, with a particular emphasis on proctitis and other GI issues. Eligible studies are quantitative in design, encompassing cross‐sectional, cohort, case–control studies, and clinical trials. Only articles published in English and available up to October 2024 are considered. Studies addressing other gastric issues, qualitative research, case series, letters to the editor, commentaries, reviews, and abstract‐only publications are excluded. Additionally, full‐text availability is a prerequisite for systematic review.

### 2.2 Search Strategy

The literature search was conducted using three primary electronic databases: PubMed, Embase, and Web of Science, encompassing studies from each database's inception through October 2024. To ensure comprehensive coverage of relevant studies, a combination of keywords and Medical Subject Headings (MeSH) terms related to Mpox and GI manifestations (e.g., proctitis and other GI issues) was utilized. The detailed search strategy for each database is documented in Table S2 to ensure both transparency and reproducibility in the inclusion of literature.

### 2.3 Screening and Data Extraction

The screening and data extraction process followed a structured approach, utilizing the software tool Nested Knowledge. Screening occurred in two stages: initially, titles and abstracts were reviewed to exclude studies that did not meet the inclusion criteria. The second stage involved a thorough full‐text review to confirm the eligibility of the remaining articles. Both stages were independently conducted by two reviewers to reduce potential bias. Discrepancies were addressed through discussion, and if consensus could not be reached, a third reviewer was consulted for the final decision. Once the eligible studies were identified, data extraction was carried out using a structured tagging method, allowing the reviewers to systematically collect detailed information such as study design, sample size, participant characteristics, and reported outcomes.

### 2.4 Quality Assessment

The quality of the included studies was assessed using an adapted form of the Newcastle–Ottawa Scale (NOS), a commonly employed tool in prevalence research [8]. The NOS measures study quality based on three key criteria: sample size, representativeness of the sample, and the accurate identification and measurement of Mpox and GI manifestation outcomes. The quality assessment results were presented in Table S3.

### 2.5 Evidence Synthesis

The statistical R program version 4.4 was used to do the meta‐analysis [9]. The *I* ^2^ statistic was used to assess the heterogeneity among the studies [10]. A leave‐one‐out sensitivity analysis was carried out, which entailed methodically removing each research study to examine its effect on the aggregate prevalence estimate, in order to gauge the robustness of the pooled data. Using a Doi plot and the LFK index value, publication bias was evaluated [11].

## 3 Results

### 3.1 Literature Search

**FIGURE 1:**
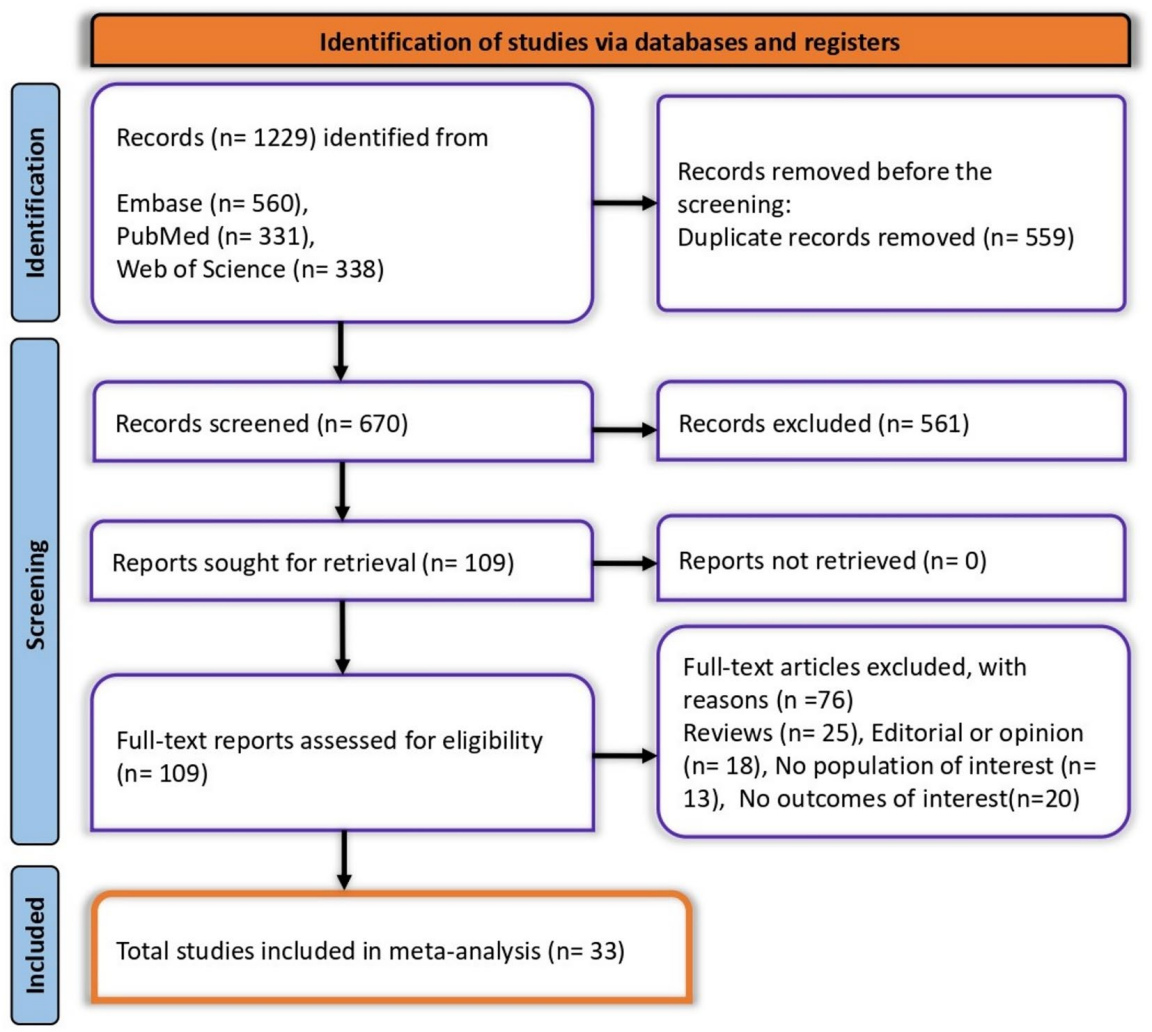
PRISMA flowchart showing the studies selection process.

A comprehensive search across multiple databases, including Embase (*n* = 560), PubMed (*n* = 331), and Web of Science (*n* = 338), yielded a total of 1229 studies. After removing 559 duplicate entries, 670 unique studies remained for screening. During the initial screening phase, 561 studies were excluded based on relevance. Subsequently, 109 full‐text reports were assessed for eligibility, with 76 being excluded due to various reasons, including reviews (*n* = 25), editorials or opinion pieces (*n* = 18), lack of population of interest (*n* = 13), and absence of relevant outcomes (*n* = 20). In the end, 33 studies were included in the final meta‐analysis (Figure 1).

### 3.2 Summary of Study Characteristics

The characteristics of the studies included in this analysis encompass a range of countries and study designs. Among the 33 studies, designs varied, with cohort studies [12, 13, 14, 15, 16, 17, 18, 19, 20, 21, 22, 23, 24, 25, 26, 27, 28, 29, 30, 31, 32, 33, 34, 35] being the most common, followed by cross‐sectional studies [36, 37, 38, 39, 40, 41, 42] and a smaller number of case–control studies [43]. These studies were conducted across diverse geographical locations, including the United States, Spain, the United Kingdom, France, Italy, Argentina, Portugal, Belgium, Mexico, Brazil, Nigeria, Zaire (Democratic Republic of the Congo), and Peru, reflecting a broad international scope of Mpox research. The mean or median age of participants in the included studies ranged from 6.9 to 40 years, with most studies focusing on adult populations (30–40 years old). The majority of the Mpox cases were men (over 95% in most studies), emphasizing the disproportionate impact of the outbreak among MSM. The clinical outcomes of interest were predominantly related to GI and anogenital manifestations, with proctitis, anorectal pain, rectal bleeding, diarrhea, and nausea/vomiting frequently reported across studies. Proctitis was the most common GI manifestation, appearing in multiple studies with varying prevalence rates. Some studies also documented additional symptoms such as oropharyngeal ulcers, odynophagia, and abdominal pain. The sample sizes of Mpox patients varied significantly across studies, ranging from 11 to 1472 patients, reflecting differences in study scale and data collection methods. Diagnosis of Mpox was predominantly confirmed through PCR testing from skin, genital, rectal, or oropharyngeal samples, although a few earlier studies utilized electron microscopy, viral culture, and serological assays.

### 3.3 Meta‐Analysis

#### 3.3.1 Prevalence of Proctitis Among Mpox

**FIGURE 2:**
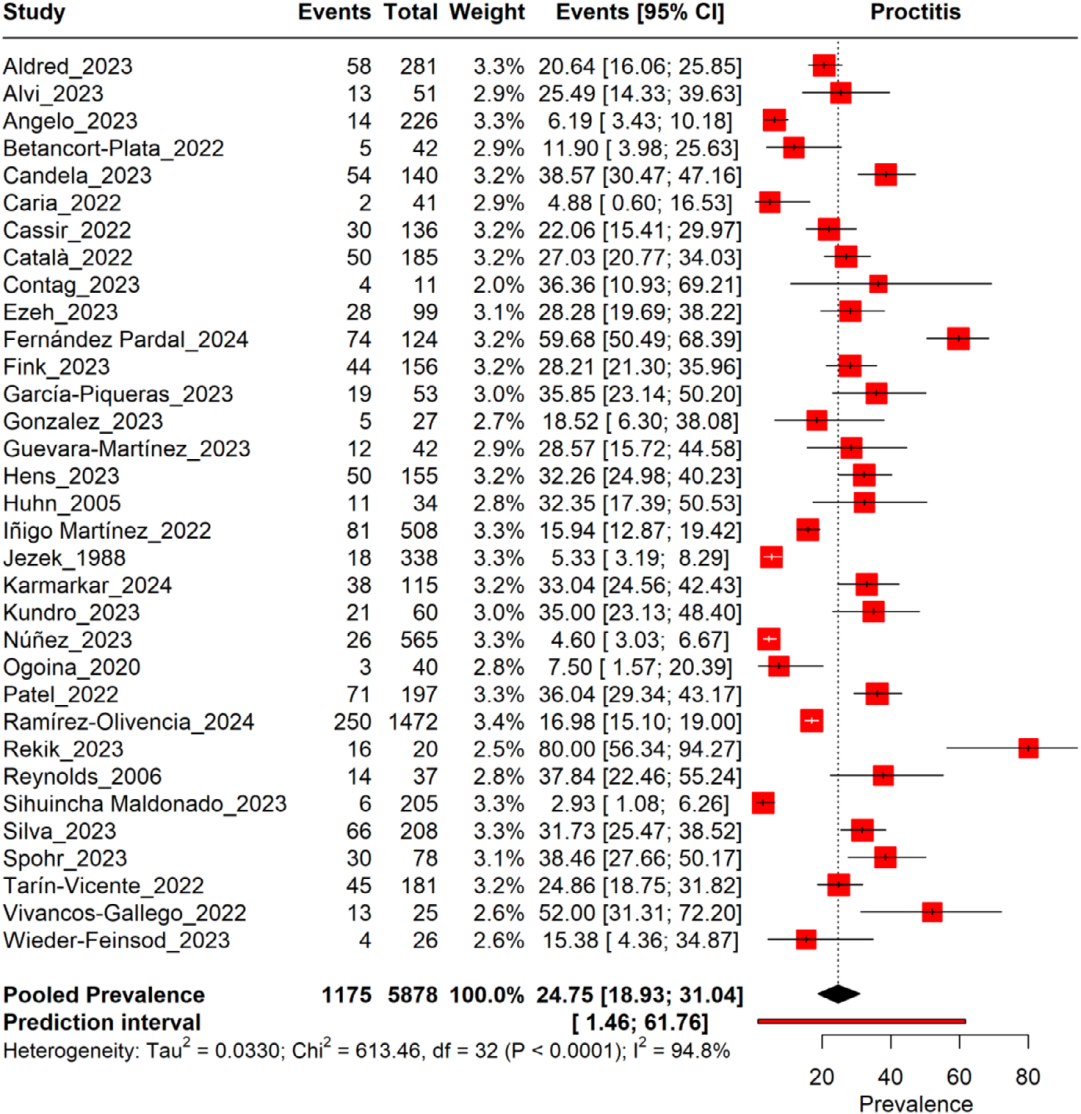
Forest plot illustrating the prevalence of proctitis conditions among monkeypox.

The pooled prevalence of Proctitis in Mpox patients across 33 studies, with a total sample size of 5878, is estimated to be 24.75% (95% CI: 18.93%–31.04%), with heterogeneity observed (*I* ^2^ = 94.8%). The prediction interval is 1.46%–61.76%. The prevalence rates vary widely across the included studies, ranging from 4.60% to 59.68% (Figure 2). A leave‐one‐out sensitivity analysis revealed that no individual study significantly influenced the pooled results (Figure S4).

#### 3.3.2 Prevalence of Other GI Among Mpox

**FIGURE 3:**
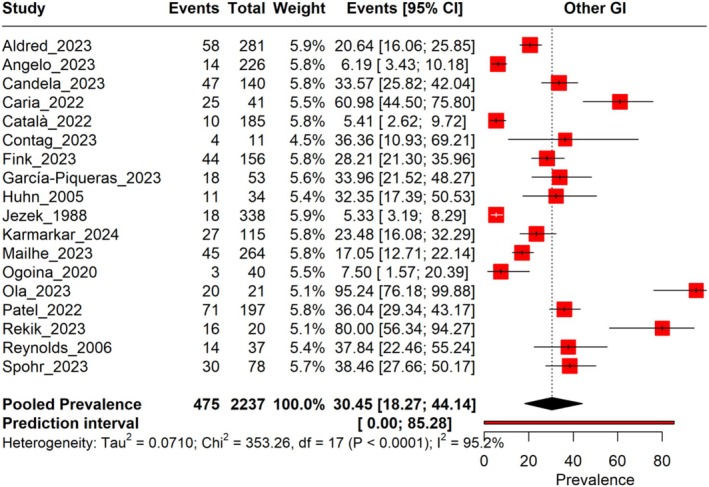
Forest plot illustrating the prevalence of other GI conditions among monkeypox.

The pooled prevalence of Other GI symptoms in Mpox patients across 18 studies, with a total sample size of 2237, is estimated to be 30.45% (95% CI: 18.27%–44.14%), with significant heterogeneity observed (*I* ^2^ = 95.2%). The prediction interval ranges from 0.00% to 85.28%, indicating substantial variability in prevalence estimates across different study settings. The prevalence rates vary widely among the included studies, ranging from 5.33% to 95.24% (Figure 3). A leave‐one‐out sensitivity analysis revealed that no individual study significantly influenced the pooled results (Figure S5, Table 1).

**TABLE 1:** Summary characteristics of included studies.

Study	Country	Study design	Mean age	Male %	Outcomes GI	No. of Mpox patients	Diagnosis of MPox
Aldred, 2023 [44]	United States	Cohort study	35	96.4	Anorectal—58	281	Mpox diagnosis confirmed through polymerase chain reaction (PCR) testing
Alvi, 2023 [43]	United States	Case–control study	34.5 (median)	89	Proctitis—13	51	Based on clinical symptoms
Angelo, 2023 [36]	18 GeoSentinel sites across 15 countries	Cross‐sectional study	37 (median)	100	Diarrhea—13 Abdominal pain—1	226	PCR from skin lesions, serum, or other specimens
Betancort‐Plata, 2022 [12]	Spain	Cohort study	40 (median)	100	Proctitis—5	42	PCR testing
Candela, 2023 [13]	Italy	Cohort study	37	98	Anal lesion—47 Proctitis—54	140	Diagnosed by PCR testing from various swab samples
Caria, 2022 [37]	Portugal	Cross‐sectional study	37.2	97.6	Proctitis—2 Anogenital—25	41	Diagnosed via PCR testing (NAAT) from skin or mucosal lesions
Cassir, 2022 [14]	France	Cohort Study	36 (median)	97.8	Proctitis—30	136	Real‐time PCR testing of skin, genital, rectal, or pharyngeal swabs
Català, 2022 [38]	Spain	Cross‐sectional Study	38.7	100	Proctalgia or proctitis—40 Oral ulcer—10	185	PCR testing for orthopoxvirus or monkeypox virus (MPXV) DNA from skin lesions
Contag, 2023 [39]	United States	Cross‐sectional study	30	63.6	Anorectal—4	11	Mpox confirmed through MPXV DNA testing using qPCR
Ezeh, 2023 [15]	United Kingdom	Cohort study	NA	NA	Proctitis—28	99	Mpox diagnosis confirmed via positive PCR testing for Mpox
Fernández Pardal, 2024 [16]	Argentina	Cohort study	31.5	99.2	Proctitis—74	124	Diagnosed via PCR testing from lesions or scabs
Fink, 2023 [17]	United Kingdom	Cohort study	35 (median)	98	Severe rectal or perianal pain—44	156	Mpox confirmed via MPXV DNA detected from clinical samples using PCR
García‐Piqueras, 2023 [40]	Spain	Cross‐sectional study	36	98	Odynophagia—18 Proctitis—19	53	All are Mpox case
Gonzalez, 2023 [41]	PuertoRico	Cross‐sectional study	32 (median)	100	Proctitis—5	27	Mpox confirmed in patients who received tecovirimat treatment
Guevara‐Martínez, 2023 [18]	Spain	Cohort study	39 (median)	100	Proctitis—12	42	Confirmed by PCR testing of skin or rectal swabs
Hens, 2023 [19]	Belgium	Cohort Study	39	100	Proctitis—50	155	Confirmed by PCR testing
Huhn, 2005 [20]	United States	Cohort study	26 (median)	53	Nausea and vomiting—11	34	PCR, viral culture, electron microscopy, and immunohistochemistry
Iñigo Martínez, 2022 [21]	Spain	Cohort study	35 (median)	99	Proctitis—81	508	Confirmed by real‐time PCR on vesicular lesion specimens
Jezek, 1988 [22]	Zaire (Democratic Republic of the Congo)	Cohort study	6.9	53.8	Vomiting, diarrhea, dehydration—81	338	Electron microscopy, viral culture, and serological assays (ELISA, HAI, RIA)
Karmarkar, 2024 [42]	United States	Cross‐sectional study	36	89.6	Rectal pain/tenesmus/proctitis—38 Rectal bleeding—27	115	Confirmed via PCR testing
Kundro, 2023 [23]	Argentina	Cohort Study	34	96.47	Proctitis—21	60	Confirmed case of mpox was defined as a positive real time PCR of eitherskin lesions or anal/oropharynx swab samples
Núñez, 2023 [24]	Mexico	Cohort study	34 (median)	97.2	Proctitis—26	565	NA
Ogoina, 2020 [25]	Nigeria	Cohort study	32 (median)	77.5	Nausea and vomiting—3	40	Clinical evaluation and confirmed by laboratory diagnosis
Patel, 2022 [26]	United Kingdom	Cohort Study	38 (median)	100	Rectal pain or pain on defecation—71	197	PCR from skin, genital, or mucocutaneous lesions
Ramírez‐Olivencia, 2024 [27]	Spain	Cohort study	38.6	99	Proctitis—250	1472	Diagnosed via PCR testing for MPXV
Rekik, 2023 [28]	France	Cohort Study	33 (median)	100	Anal problem—16	20	Diagnosed via PCR testing for MPXV on skin and/or oropharyngeal swabs
Reynolds, 2006 [29]	United States	Cohort study	NA	46.8	Gastrointestinal—14	37	PCR, MPXV isolation, or immunohistochemistry
Sihuincha Maldonado, 2023 [30]	Peru	Cohort study	32 (median)	98.5	Proctitis—6	205	Diagnosed by PCR testing of skin lesions
Silva, 2023 [31]	Brazil	Cohort study	33 (median)	96.2	Proctitis—66	208	PCR testing of skin lesion, anal, or oropharynx swabs
Spohr, 2023 [32]	Germany	Cohort Study	38	98.71	Proctitis and rectal bleeding—30	78	NA
Tarín‐Vicente, 2022 [33]	Spain	Cohort study	37 (median)	97	Proctitis—45	181	Laboratory‐confirmed monkeypox (PCR testing on lesion, anal, and oropharynx swabs)
Vivancos‐Gallego, 2022 [34]	Spain	Cohort study	39.5	100	Proctitis—13	25	Diagnosed through laboratory‐confirmed MPXV DNA detection, mostly from skin or genital swabs.
Wieder‐Feinsod, 2023 [35]	Israel	Cohort study	34.5	100	Proctitis—4	26	Confirmed by PCR testing

#### 3.3.3 Subgroup Analysis

##### 3.3.3.1 Study Design

The pooled prevalence of Proctitis in Mpox patients across 33 studies, with a total sample size of 5878, is estimated to be 24.75% (95% CI: 18.93%–31.04%), with heterogeneity (*I* ^2^ = 94.8%) and a wide prediction interval of 1.46%–61.76%. When stratified by study design, cohort studies had the prevalence at 25.87% (95% CI: 18.73%–33.69%), case–control studies at 25.49% (95% CI: 13.33%–39.33%), and cross‐sectional studies with the prevalence at 20.62% (95% CI: 8.68%–35.70%) (Table 2).

**TABLE 2:** Subgroup analysis of proctitis manifestation condition among monkeypox by study design.

Subgroup	Type	No. of studies	Sample size	Events	Pooled prevalence (%)	Heterogenity (*I* ^2^)
Study design	Cohort study	25	5169	1030	25.87	95.6
	Cross‐sectional study	7	658	132	20.62	91.5
	Case–control	1	51	13	25.49	NA
	Overall	33	5878	1175	24.75	94.8

##### 3.3.3.2 Publication Bias

The publication bias assessment using DOI plots reveals significant asymmetry, with LFK index values of 2.77 (Figure S6) and 2.8 (Figure S7). Typically, LFK index values beyond ±2 indicate major asymmetry, suggesting the presence of substantial publication bias in the included studies.

## 4 Discussion

This systematic review and meta‐analysis provide robust evidence regarding the prevalence and clinical significance of proctitis and other GI manifestations in Mpox patients. The pooled prevalence of proctitis was estimated at 24.75% (95% CI: 18.93%–31.04%), highlighting its frequent occurrence among individuals diagnosed with Mpox. Similarly, other GI symptoms, including diarrhea, rectal bleeding, nausea, vomiting, and abdominal pain, were prevalent in 30.45% (95% CI: 18.27%–44.14%) of cases. These findings emphasize the importance of recognizing GI involvement in Mpox to enhance clinical management and improve patient outcomes. The significant heterogeneity observed in prevalence estimates (*I* ^2^ = 94.8% for proctitis; *I* ^2^ = 95.2% for other GI symptoms) underscores the variability in Mpox presentations across different populations and study designs. This variation may stem from differences in diagnostic methods, sample sizes, geographic regions, and patient demographics. Despite these differences, the consistent documentation of GI involvement across multiple studies supports the notion that these symptoms are not incidental but rather integral to the clinical spectrum of Mpox.

The findings of this meta‐analysis align with prior research while expanding the understanding of GI manifestations in Mpox. Previous studies reported that proctitis, nausea, vomiting, and diarrhea were frequently observed in Mpox patients, though inconsistently documented across studies [45]. Similarly, earlier reviews underestimated proctitis prevalence, reporting rates as low as 11%, whereas newer data suggest a significantly higher burden. Our meta‐analysis, incorporating recent findings, estimates the pooled prevalence of proctitis at 24.75% (95% CI: 18.93%–31.04%), reinforcing its role as a significant clinical feature of Mpox [46]. Prior reviews primarily focused on dermatologic and systemic symptoms, with limited discussion on GI involvement [47]. In contrast, this study provides a focused quantitative synthesis of Mpox‐related GI symptoms, underscoring their diagnostic and therapeutic importance. The high heterogeneity observed across studies (*I* ^2^ = 94.8%) aligns with previous research and reflects variations in study populations and methodologies. Subgroup analysis also found that cohort studies reported a higher prevalence of proctitis (25.87%) compared to cross‐sectional studies (20.62%). While prior studies suggested that Mpox‐associated proctitis may be mistaken for STIs, this analysis highlights its distinct pathophysiology. Given the significant prevalence of GI symptoms in Mpox, further research is needed to elucidate underlying mechanisms and inform clinical management strategies.

Sensitivity analyses confirmed the stability of our results, with no single study significantly influencing the overall prevalence estimates. The leave‐one‐out method demonstrated that the prevalence estimates remained consistent across different study exclusions, reinforcing the reliability of our findings. However, the presence of publication bias (LFK index > 2.77) suggests potential underreporting of negative or nonsignificant results, which could influence the observed effect sizes. Addressing this bias in future research will be crucial for refining estimates of Mpox‐associated GI manifestations.

These findings have important clinical and public health implications. Given the substantial burden of proctitis and other GI symptoms, healthcare providers should incorporate routine GI assessments into the diagnostic workup of Mpox patients, particularly in high‐risk populations [48]. Early recognition and management of these symptoms can improve patient outcomes and reduce complications such as secondary infections, dehydration, and prolonged hospitalization [49]. Furthermore, clinicians should be aware of the high co‐occurrence of Mpox and STIs, necessitating comprehensive screening protocols to ensure appropriate treatment strategies [50]. The study also underscores the need for targeted public health interventions, particularly in communities disproportionately affected by Mpox. Public health campaigns should emphasize awareness of GI symptoms and encourage early healthcare‐seeking behaviors [51]. Additionally, further research is required to elucidate the pathophysiological mechanisms underlying Mpox‐associated GI symptoms, including the role of direct viral invasion, immune‐mediated inflammation, and co‐infections.

Despite its strengths, this study has several limitations. First, the high heterogeneity in prevalence estimates limits the generalizability of findings across different settings. Second, most included studies relied on observational designs, which preclude causal inferences regarding Mpox and GI symptoms. Third, variations in diagnostic criteria for proctitis and GI symptoms across studies may have contributed to inconsistencies in prevalence estimates.

Future research should prioritize prospective cohort studies to establish temporal relationships between Mpox infection and GI manifestations. Moreover, studies investigating the role of sexual transmission pathways in Mpox‐related proctitis could provide valuable insights into disease pathogenesis. Finally, standardized diagnostic criteria for Mpox‐associated GI symptoms are needed to improve comparability across studies and enhance clinical decision‐making.

## 5 Conclusion

This systematic review and meta‐analysis highlight the substantial burden of proctitis and GI symptoms in Mpox patients, with prevalence rates of 24.75% and 30.45%, respectively. These findings underscore the need for heightened clinical awareness and comprehensive management strategies to address GI involvement in Mpox. Given the ongoing global Mpox outbreaks, future research should focus on elucidating disease mechanisms, refining diagnostic criteria, and developing targeted interventions to mitigate the impact of Mpox‐associated GI complications.

## Ethics Statement

The authors have nothing to report, as there were no human participants involved in this study.

## Consent

The authors have nothing to report, as this study does not involve any individual person's data in any form.

## Conflicts of Interest

The authors declare no conflicts of interest.

## Supporting information


**Table S1.** PRISMA checklist.
**Table S2.** The adjusted search terms as per searched electronic databases.
**Table S3.** Modified Newcastle–Ottawa Scale for the quality assessment of included studies.
**Figure S4.** Leave‐one‐out analysis representing results of proctitis.
**Figure S5.** Leave‐one‐out analysis representing results of other GI issues.
**Figure S6.** Publication bias assessment using DOI plots of proctitis associated among monkeypox population.
**Figure S7.** Publication bias assessment using DOI plots of other GI issues associated among monkeypox population.

## Data Availability

All data generated or analyzed during this study are included in this published article (and its Supporting Information files).
